# Mobile dentistry and digital dentures in aged care placements: dental students’ experiences and perceptions

**DOI:** 10.3389/froh.2026.1923430

**Published:** 2026-09-16

**Authors:** Archana Pradhan, Zakia Sultana Happy, Rahena Akhter, Arash Rudman, Shilpi Ajwani

**Affiliations:** 1 School of Dentistry, Faculty of Medicine and Health, The University of Sydney, Sydney, NSW, Australia; 2 Australian Research Centre for Population Oral Health, College of Health, Adelaide University, Adelaide, SA, Australia; 3 Centre for Education and Research on Ageing (CERA), Department of Geriatric Medicine, Concord Repatriation Hospital, Sydney, NSW, Australia

**Keywords:** aged care placements, dental workforce, digital dentures, interdisciplinary education, mobile dentistry, prevention

## Abstract

**Background:**

Accessing dental care for residents of Residential Aged Care Facilities (RACFs) is challenging especially for conventional prosthodontic care. This burden is exacerbated by multi-level barriers spanning individual, professional, institutional, and health-system domains. Strengthening alignment between innovations in technology, dental education and service delivery is crucial in addressing the increasing oral health needs of the ageing population. This paper reports on dental students' experiences and perceptions of interdisciplinary aged care placements, incorporating mobile dentistry and exposure to digital denture workflows within RACFs.

**Methods:**

A cross-sectional mixed-methods educational evaluation was conducted using a semi-structured questionnaire completed by dental students who participated in RACF placements (2025–2026). The placements provided onsite dental care using portable dental equipment under the supervision of a Specialist in Special Needs Dentistry and a dental prosthetist. Quantitative data were analyzed descriptively using frequencies and percentages, and qualitative responses were analyzed inductively using thematic analysis.

**Results:**

Students reported positive perceptions regarding interdisciplinary learning and collaboration, application of digital denture workflows and increased awareness of oral healthcare inequities among frail older adults. They also identified barriers associated with outreach oral healthcare delivery within RACFs, including patient frailty, cognitive impairment, communication difficulties, and logistical challenges associated with mobile dentistry and digital workflows.

**Conclusion:**

This preliminary evaluation suggests interdisciplinary RACF placements integrating mobile dentistry and digital denture workflows can provide valuable experiential learning opportunities and support students' understanding of geriatric oral healthcare, mobile dentistry, and digital dentistry workflows.

## 1 Introduction

Population ageing is a growing public health challenge worldwide, with older adults experiencing complex medical, functional, and oral health needs (1, 2). Oral health is frequently poorly managed in Residential Aged Care Facilities (RACFs), despite its important role in the overall health and quality of life (2). Notably, RACF residents face a high burden of health-related vulnerability, including frailty and severe impairment in basic activities of daily living (3). Consequently, preventable oral diseases (dental caries, periodontal disease, and denture-related conditions) remain highly prevalent among them (1, 2). These untreated problems can contribute to avoidable hospitalizations and increased healthcare costs (4). Recent studies underscore further challenges this population faces in accessing dental services, stemming from multi-level barriers across individual, professional, institutional, and health systems, including shortages of competent trained workforce (5), funding limitations (1), and poor collaboration between dental and aged care services (2, 6). These structural and system-level challenges highlight the need for sustainable, preventive oral health care models for older adults living in residential care settings.

Over 25 years ago, Lee and Thomas stated that “as our population ages further, portable and mobile dentistry will be a necessity, not a luxury” (7). Broadly, mobile dentistry is an umbrella term for different mobile systems, such as equipped vans or domiciliary dental services, that reach people who cannot access services themselves (8). Domiciliary dental service models can be a practical approach to the unmet oral health needs of the elderly (2, 9, 10). Benefits are achieved through the delivery of on-site preventive and restorative care, which reduces barriers such as travel time, transportation difficulties, and anxiety associated with conventional dental visits (1). However, the uptake of this service model by dentists remains low. The implementation of the Reach-OHT model (2013) in Sydney, Australia, is an example of a successful domiciliary dental program in which oral health therapists conducted oral health assessments, and on-site treatment was provided by participating private dentists using portable dental equipment (10). The program showed that preventive and minimally invasive oral health care can be effectively delivered within RACFs, reducing financial, logistical, and access-related barriers. The large proportion of residents requiring further dental care following oral health assessments successfully completed their treatment plans through structured referral pathways and coordinated service delivery. Another domiciliary service model in Quebec, Canada was limited to preventive services, fillings, extractions, and denture repairs, without the use of portable x-ray units (11).

In addition to service innovations, aged-care clinical placements for dental and oral health students have the potential to strengthen students' preparedness for providing care in Geriatric and Special Needs Dentistry (12). An example is the University of Newcastle program introduced in 2009, where students were placed across 17 RACFs for weekly sessions providing oral health education and daily care support (13, 14). These models suggest that direct aged care exposure may strengthen students' knowledge, preparedness, and confidence in caring for older adults while supporting preventive oral healthcare in residential care settings (13, 14). However, such initiatives remain limited and are inconsistently integrated into dental curricula, and workforce participation in domiciliary oral health services remains low (6, 12).

With advances in digital technology, dental curricula should critically engage with digital health technologies and their applications in patient care (15). However, the education system has been unable to implement a well-developed module for undergraduate dental curricula, and students often graduate with inadequate skills in providing care for frail older adults (16, 17). Dental practitioners themselves lack confidence in providing dental care to older people, especially those with cognitive impairment due to inadequate training and experience, yet many do not take advantage of available online training (18). Addressing the impact of an ageing population on oral health and oral healthcare systems should be a public health priority (19). Innovative models of care including digital workflows should be included in the dental curriculum to prepare the dental workforce to meet the complex dental treatment needs of the elderly (16, 17, 20). Dental practitioners should be equipped to be both globally competent and responsive to the growing needs of the communities they serve within the health systems. Strengthening alignment between dental education and service delivery is crucial in addressing the challenging oral health needs of the ageing population.

In response to the health-related challenges faced by aging populations, the World Health Organisation (WHO) developed the Decade of Healthy Ageing global action (2020–2030), with ten priorities for action, one of which is to align health systems with the needs of older people where “older adults get the health care they need—where and when they need it” (21). Despite such global action plans and national policy frameworks such as the National Oral Health Plan 2015–2024 (22) and the Royal Commission into Aged Care Quality and Safety (23) implementation remains constrained by workforce shortages, fragmented service delivery, and funding limitations.

This brief research report describes an interdisciplinary educational model introduced at RACFs in Sydney, Australia between 2025 and 2026. The report aimed to explore dental students' self-reported experiences and perceptions of aged care placements involving preventive oral healthcare, mobile dentistry, interdisciplinary collaboration, and exposure to digital denture workflows under the supervision of a Specialist in Special Needs Dentistry and a dental prosthetist. Specifically, it examined students' perceived learning experiences, confidence in digital scanning, barriers encountered during the placement, and views on integrating aged care placements and digital denture training into dental curricula.

## 2 Methods

At The University of Sydney, School of Dentistry, regular feedback is provided to and collected from students as part of their routine clinical placement evaluation and educational quality improvement processes. All students provide consent at enrolment for the use of their feedback for educational improvement purposes. At the end of each semester (in July for Semester 1 and November for Semester 2) all students are asked to complete Units of Study Surveys (USS). The USS feedback is confidential and helps the study coordinators identify what was done well and what needs improvement. Both positive and constructive feedback are welcomed. Despite positive feedback from students during placements there was no mention of experience in Aged Care in semester 1 in 2025. By the time the students completed their USS, their short placement experience in aged care was overshadowed or buried under the other clinical placements in other specialties. To obtain more detailed insight into this interdisciplinary educational model and explore students' perceptions and experiences, all students who completed the RACF placement between October 2025 and April 2026 were invited to participate in an additional evaluation questionnaire. All 36 eligible students who completed the RACF placement during the study period were invited to participate. Participants included Doctor of Dental Medicine (DMD) Year 4 students rostered in aged care placements. A group of four students visited a RACF once a week, with two sessions per semester. Under the supervision of a Specialist in Special Needs Dentistry (SND) and a dental prosthetist, the sessions incorporated mobile dentistry, preventive oral healthcare, and digital denture workflows. The services included prevention and management of oral conditions, including oral health screening, toothbrushing with modified toothbrushes, fluoride application, radiographs, silver fluoride, atraumatic restorative treatment (ART), extractions, digital dentures, and denture care.

This report uses a cross-sectional mixed-methods educational evaluation design combining descriptive quantitative analysis with qualitative thematic analysis. The questionnaire was developed by the research team, informed by relevant literature and placement objectives, and reviewed by the supervisory team for clarity and relevance (24). Quantitative and qualitative data were collected at one time point following students' participation in the RACF placement using a semi-structured questionnaire. Quantitative data included student demographics, prior experience with digital denture scanning, self-reported engagement with scanning activities (number of scans and time required), and ratings of instructional effectiveness, scanner usability, time efficiency, confidence for independent scanning, and perceived curriculum relevance using a five-point Likert scale (1–5). Qualitative items explored changes in student interest in aged care dentistry, barriers and challenges to implementing digital denture workflows in RACFs, facilitators for wider adoption, and opinions on integrating digital denture training and aged care placements into the core dental curriculum.

The questionnaire was distributed via Microsoft Office Forms at the end of each placement with two reminders sent at two-week intervals. Participation in the questionnaire was voluntary. As the report involved anonymous educational evaluation data with minimal risk to participants, a waiver of consent was obtained from the University of Sydney Research Integrity and Ethics Administration (2026/HE000543).

Quantitative data were exported to Microsoft Excel and analysed descriptively using frequencies and percentages. Percentages were calculated using the number of valid responses for each item. The number of scans completed and perceived curriculum relevance were analysed using the full sample of respondents. Scanning-experience items were analysed among students who completed at least one scan. Initial coding of the qualitative responses was conducted by ZSH using Microsoft Excel. AP led the analysis and interpretation by reviewing the codes and candidate themes with ZSH, but did not independently recode the responses. Minor differences concerning the wording, grouping of codes and candidate themes were resolved by revisiting the original participant responses and through discussion between ZSH and AP until mutual agreement was reached. No substantial interpretive disagreements arose. AR subsequently reviewed the candidate themes and supporting quotations. The analysis and final themes were then discussed during regular team meetings involving AP, ZSH, AR, RA and SA. Candidate themes were reviewed and refined through team discussion until consensus was reached, thereby strengthening the credibility and consistency of the analysis. The broader team discussion informed the refinement and presentation of the four final themes but did not involve independent recoding of the responses. Qualitative responses were analysed using an inductive thematic analysis approach following Braun and Clarke, involving repeated reading of the data, initial coding, and iterative theme development (25). Coding was primarily semantic and focused on the explicit meanings of the students' responses rather than inferred latent meanings. Codes were applied to meaningful segments comprising phrases, sentences or complete responses, depending on how each idea was expressed. The complete response was considered to preserve context, and codes were generated from meaning rather than individual word or term frequency. The four final themes emerged inductively from the participants’ responses rather than from a predetermined analytical framework. As the qualitative data consisted of open-ended questionnaire responses from a predefined student cohort rather than sequential qualitative interviews, formal data saturation was not used to determine the sample size. Finally, findings from the quantitative and qualitative analyses were interpreted together to better understand students' learning experiences and perceptions of the RACF placement. This also helped evaluate the educational relevance of integrating mobile dentistry and digital dentistry exposure within aged care placements.

## 3 Results

### 3.1 Participant characteristics

A total of 28 out of 36 students (77.7%) responded to the questionnaire (Table 1). They were aged between 21 and 34 years, with males representing 75.0% of the participants. 23 students had the opportunity to complete at least one scan, with 15 students completing 1–2 scans and eight students completing 3–5 scans. Five students reported no opportunity to perform digital denture scanning during the placement period.

**Table 1:** Student characteristics.

Characteristic	*n* (%)
Age group	
21–34 years	28 (100)
Gender	
Male	21 (75.0)
Female	7 (25.0)
Prior experience with digital denture scanning	
Yes	6 (21.4)
No	22 (78.6)

### 3.2 Student experiences and perceptions of digital denture workflows

Among the 28 respondents, 15 students had the opportunity to complete one or two scans (53.6%), while eight (28.6%) completed 3–5 scans and five (17.9%) completed no scans (Table 2). Of the 23 students who completed at least one scan, 17 (73.9%) reported completing a scan in under 10 min, while five students reported scan times of 10–20 min and one reported taking more than 20 min. Among these 23 students, 21 (91.3%) agreed or strongly agreed that the intraoral scanning demonstration was effective, 18 (78.3%) considered that handling the intraoral scanner within the RACF environment was manageable, and 18 (78.3%) considered that the time required for scanning was acceptable. However, confidence in independently performing intraoral scanning procedures was lower. 56.5% of students agreed or strongly agreed that they felt comfortable scanning independently following placement exposure. Nevertheless, more than two-thirds of the students (71.4%) supported inclusion of digital denture workflows in dental education curricula. The full distribution of responses across all five Likert-scale categories is presented in Supplementary Table S1.

**Table 2:** Student experiences and perceptions of RACF placements and digital denture workflows (*n* = 28).

Variables	*n* (%)
Scanning exposure (*n* = 28)	
None	5 (17.9)
1–2 scans	15 (53.6)
3–5 scans	8 (28.6)
Time required to complete a scan—students completing ≥1 scan (*n* = 23)	
<10 min	17 (73.9)
10–20 min	5 (21.7)
>20 min	1 (4.3)
Perceptions (Agree/Strongly Agree)- students completing ≥1 scan (*n* = 23)	
Demonstration of intraoral scanning was effective	21 (91.3)
Handling the intraoral scanner in the aged care setting was easy	18 (78.3)
Time required to complete intraoral scanning was acceptable	18 (78.3)
Comfortable performing intraoral scanning independently after training	13 (56.5)
Curriculum inclusion (*n* = 28)	
Digital denture scanning should be included in the dental curriculum	20 (71.4)

Perception items were measured using a five-point Likert scale ranging from strongly disagree (1) to strongly agree (5). Percentages were calculated using the number of valid responses for each item. Scanning-experience items used a denominator of 23, whereas the curriculum-inclusion item used the full sample of 28 respondents.

While the students reported that the RACF placement improved their understanding of oral healthcare needs among frail older adults, they also reported increased awareness of barriers associated with oral healthcare delivery within aged care settings.

### 3.3 Qualitative findings

Four major themes were identified: experiential and transformative learning in aged care; interdisciplinary learning and collaboration in digital workflow; barriers and opportunities in aged care placements; support for integration of digital dentures in aged care placements within graduate dental education.

#### 3.3.1 Theme 1. Experiential and transformative learning in aged care

Most students described these placements as “eye opening” and “insightful” experiences that provided “a realistic snapshot”, significantly expanding their understanding of the vulnerabilities, limitations, and growing unmet oral health needs of older adults and broadened their scope of dentistry within aged care facilities.

“I saw first-hand the need for it and the demand for it. I feel like it is an under-serviced demographic, which will benefit tremendously from associated programs.” [Student 9]

“Helped me see how vulnerable such individuals are and therefore how important it is for us to care for such ones.” [Student 2]

“It's sad that their oral health is neglected so much so I’d love to help improve it.” [Student 11]

Interaction with the residents highlighted the role of Special Needs Dentistry, the need for triage, and individualized treatment planning for medically complex cases.

“Different disciplines involve different approaches… it is important to know how to triage these cases and have an idea of their management.” [Student 12]

Several students additionally reported that the placement broadened their understanding of mobile dentistry and future professional roles within aged care settings.

“Introduced a new avenue of dentistry which I can explore in my practice.” [Student 15]

“Provided insight into how dentists can help this demographic; inspired me to consider outreach programs in aged care centres.” [Student 4]

For one student it “decreased interest in aged care dentistry due to complexity and patient management demands, though compassion increased”. For two students, it increased interest only in digital scanning.

#### 3.3.2 Theme 2. Interdisciplinary learning and collaboration in digital workflows

The students highlighted several ways in which interdisciplinary collaboration and digital denture workflows enhanced their learning experiences during the RACF placement. They reported that observing and engaging in teamwork with Oral Health Therapy (OHT), Doctor of Dental Medicine (DMD) and Doctor of Clinical Dentistry (DCD) students, an experienced dental prosthetist and a Specialist in Special Needs Dentistry contributed significantly to their understanding of the scope of practice of each dental practitioner. Exposure to different professional roles and approaches enhanced their understanding of patient-centred care and broadened their appreciation of collaborative practice in managing complex cases.

“Exposure to prosthetists; insight into digital workflows and philosophical approach to elderly care.” [Student 8]

The integration of digital denture workflows was widely perceived as a key facilitator of learning. The students noted that the portability of intraoral scanning technology enabled on-site treatment, reducing the need for residents to travel to fixed-chair dental clinics for replacement of lost or damaged dentures, thereby minimising stress and discomfort. Digital workflows were described as more efficient and streamlined compared to traditional methods, particularly in resource-limited aged care settings.

“It is much faster especially for aged care centres where there may not be a lot of equipment.” [Student 4]

“Much more comfortable for the patient and can be faster than conventional methods in the right hands. Even if dentures were adequate, they were scanned for record keeping so if they get lost or misplaced, it's a simple process of reprinting the denture.” [Student 25]

Importantly, the students emphasised that the combination of technology and guided supervision supported experiential learning. Demonstrations by experienced clinicians, along with opportunities to observe and participate in scanning procedures, enhanced students' confidence and understanding of digital workflows. Many highlighted that structured guidance made the technology more accessible and easier to apply in practice.

“Demonstration was helpful; scanning is simple with proper guidance.” [Student 17]

#### 3.3.3 Theme 3. Barriers and opportunities in aged care placements

Although students reported positive educational experiences overall, several clinical, technical, and organisational challenges associated with RACF placements were identified. Commonly reported barriers included patient cooperation difficulties, cognitive impairment, communication barriers, logistical issues, staffing limitations, equipment setup constraints, and restricted clinical space within RACFs. They also described technical challenges associated with digital denture workflows, particularly when conducting scans for frail older adults or for metal-based dentures within non-traditional clinical environments.

“Patient cooperation and lots of administration due to coordination required from the nursing home.” [Student 9]

“The scanning device is quite large and sometimes it does not fit patients’ mouths or makes them uncomfortable.” [Student 17]

“I thought this placement showed me the challenges that come with providing treatment to patients in aged care and provided a realistic snapshot of what limitations can be expected in this discipline.” [Student 18]

Several DMD students missed out on opportunities for hands-on treatment and digital scanning exposure due to case complexity and placement allocations during their rostered placement.

“Nobody in my group did digital scanning.” [Student 12]

“Would have appreciated doing some treatment and seeing the digital workflow.” [Student 22]

#### 3.3.4 Theme 4. Support for integration of digital dentures in aged care placements

Despite several barriers, the students consistently reported that exposure to real-world challenges enhanced their understanding of patient-centred care within aged care settings. The students strongly advocated for the inclusion of digital denture workflows and RACF placements within dental curricula, emphasising the unique opportunity to engage with high-risk, underserved populations. Many emphasised that exposure to both aged care settings and digital technologies provided a valuable and well-rounded learning experience, enabling them to develop essential competencies, including clinical judgment, adaptability, and interdisciplinary understanding. Exposure to modern, portable digital denture technology was highlighted as particularly beneficial, reinforcing skills that are increasingly relevant in contemporary dental practice. Such placements offered a unique form of experiential learning that extended beyond traditional clinical education, that were not typically available within standard clinical settings, contributing to a deeper appreciation of the social and professional responsibilities of dental practitioners.

“This should be mandatory.” [Student 23]

“RACF placements are important too; it's a different way of clinical thinking for a unique population.” [Student 25]

“I thoroughly enjoyed both of them- gave me a bit of experience in both aged care and scanning… I really appreciated the opportunity, and I think it should be in the dental curriculum or at least an option.” [Student 4]

“Strongly yes, to include digital denture training and RACF placements as a required part of the dental curriculum—essential for preparing students for future practice.” [Student 8]

“Absolutely… to gain more experience in demographics we will need to treat when we graduate. Great learning experience.” [Student 9]

“Yes, it was such a valuable experience and is like nothing else I’ve done before in dental school.” [Student 11]

The students also offered constructive recommendations to enhance the program further. Suggestions included ensuring consistent access to digital workflows, increasing hands-on scanning opportunities, and improving logistical coordination. Such enhancements, they argued, would not only optimise learning outcomes but also position the placement as a scalable, sustainable model for dental education.

“Placements should be streamlined to give students experience in seeing aged care patients as well as denture scanning. It was rewarding to see the workflow and treatment considerations for elderly patients.” [Student 15]

Overall, findings from both quantitative and qualitative analyses suggested that students perceived interdisciplinary RACF placements as valuable experiential learning opportunities integrating mobile dentistry, patient-centred care, interdisciplinary collaboration, and exposure to digital workflows within aged care settings.

## 4 Discussion

This study reports on an Australian interdisciplinary aged care placement involving dental students across various stages of learning (OHTs, DMD and DCD students) within their respective scopes of practice under the supervision of a Specialist in SND and a dental prosthetist. However, the present evaluation specifically examined the experiences and perceptions of participating DMD Year 4 students. Earlier Australian aged care placement programs, including the University of Newcastle service-learning model, primarily focused on oral health assessment, oral hygiene education and support, and engagement with residents in aged care environments (12–14). The present placement extended these earlier approaches by incorporating supervised on-site dental treatment, mobile dentistry, interdisciplinary collaboration, and exposure to digital denture workflows. Nevertheless, because the present study did not directly compare these placement models, use equivalent outcome measures, or objectively assess clinical competence, it cannot determine whether the current model produced better educational outcomes. Comparative studies using standardised measures of confidence and competence are therefore needed to evaluate the relative educational effects of these approaches.

While under the initial OHT-led program, 69.1% of the residents required dental referral following dental assessment (9), with the inclusion of DMD and DCD students, most dental treatment was provided on-site. Although the present study did not evaluate service-delivery or resident outcomes, this context illustrates the clinical environment in which the participating DMD Year 4 students completed their placements. Through this experience, students gained insight into the range of dental treatments that can be delivered within aged care settings. Direct engagement with RACF residents also strengthened their understanding of the complex oral healthcare needs and care-related challenges experienced by frail and care-dependent older adults. The students became aware of the possibilities of dental treatment in aged care settings. They reported that the RACF placement provided meaningful exposure to vulnerable older adults and increased their awareness of the unique needs and limitations of this population. Most of them were positive regarding the inclusion of digital denture workflows within RACF placements. Previous research suggests that integrating digital workflows into dental education may support technical learning, clinical reasoning, and patient-centred care (26). However, positive perceptions of the training did not uniformly translate into confidence in independent scanning with 13 of the 23 students who completed at least one scan (56.5%) agreeing or strongly agreeing that they felt comfortable scanning independently after the placement. This is likely related to the inherent learning curve associated with digital dentistry (27). The short placement period and limited opportunities for adequate hands-on practice may have further contributed to lower confidence levels. While introductory exposure is valuable, students' competency development depends on ongoing supervised practice and skill development. These findings have implications for curriculum design, suggesting that experiential placements and digital workflows should be longitudinally integrated rather than delivered as short exposures. The uneven distribution of hands-on scanning opportunities across student groups represents an important area for improvement, particularly as one student reported that “Nobody in my group did digital scanning.” Future placements could use structured rotation schedules to allocate supervised scanning opportunities more equitably where clinically appropriate. Placement activity checklists or individual learning logs could document whether each student observed, assisted with, or performed scanning and help identify students requiring additional practice. When resident complexity, consent, or case availability prevents direct clinical scanning, supplementary simulation-based sessions could provide an alternative opportunity to practise the digital workflow (26, 27). Improved coordination among clinical supervisors, RACF staff, and placement administrators may also help identify suitable residents, prepare equipment and clinical space, and allocate student roles before each session. These strategies may promote more consistent learning opportunities while ensuring that resident comfort, consent, safety, and clinical needs remain the priority.

The students also identified several challenges to implementing digital denture workflows in RACF settings, including patient factors such as cognitive impairment and limited patient cooperation. Additionally, they reported logistical and administrative constraints, such as limited space for equipment setup, and inadequate administrative support. This highlights the need to strengthen infrastructure and organisational planning when implementing digital dentistry in aged care facilities (28). Effective and successful implementation requires system-level support, proper workflow design, and coordination with RACF staff. These findings indicate that introducing digital technology alone is unlikely to ensure successful implementation. Appropriate resident selection, suitable clinical space, advance equipment preparation, adequate supervision, and clear coordination with RACF staff are also required. Pre-placement planning among clinical supervisors, facility staff, and placement administrators may help anticipate these barriers and support more efficient and resident-centred workflows.

Despite the challenges, this report highlights the value of experiential, interdisciplinary education and collaborative practice. The students recognized the importance of collaboration across professional roles, observing how OHTs, dental students, and the prosthetist work together to optimize patient care (6). These experiences reinforced the relevance of Special Needs Dentistry, clinical triage, and adaptability in clinical decision-making. Qualitative insights indicate that this exposure not only enhanced technical skills but also fostered empathy, professional responsibility, and a nuanced understanding of geriatric oral healthcare. Although most students described the placement positively, one student reported decreased interest in aged care dentistry because of the complexity of treatment and patient-management demands, despite developing greater compassion for residents. This contrasting finding indicates that exposure to aged care environments may not uniformly increase students' interest in this area of practice. Previous Australian research similarly found that some students felt overwhelmed and insufficiently prepared when entering residential aged care environments, highlighting the importance of realistic pre-placement preparation (13). Structured preparation, close supervision, and opportunities for guided reflection may help students understand and manage the clinical and emotional demands of caring for frail older adults.

However, the placement did not uniformly increase students' interest in aged care dentistry. One student reported decreased interest because of the complexity of treatment and patient-management demands, despite developing greater compassion for residents. This contrasting response demonstrates that meaningful learning and increased empathy do not necessarily lead to greater interest in pursuing aged care practice. Previous Australian research similarly found that some students felt overwhelmed and insufficiently prepared when first entering residential aged care environments (13). Frailty, cognitive impairment, communication difficulties, and unfamiliar clinical conditions may be particularly challenging for students with limited previous aged care experience. Realistic pre-placement preparation, close clinical supervision, and guided reflection may help students understand these challenges, manage their emotional responses, and develop confidence in caring for frail older adults.

The convergence of findings demonstrates a clear pattern: students not only appreciated the technical and procedural aspects of digital denture scanning but also recognised the broader clinical and social importance of providing care to an underserved population. The placement brought together contemporary digital technology, patient-centred care, and interprofessional learning and collaboration within aged care environment. Even with initial practical challenges, the students predominantly reported that the RACF placement was a highly rewarding educational experience that broadened their understanding of clinical practice and geriatric dentistry and reinforced the value of digital workflows in real-world settings. These findings support the progressive integration of aged care placements and digital denture education within dental curricula (15–17, 20, 26). However, students' positive perceptions should not be interpreted as evidence of improved clinical competence, workforce preparedness, patient outcomes, or access to care. Future research should include larger multicentre cohorts, validated evaluation instruments, objective assessments of clinical competence, and longitudinal follow-up to determine whether these educational experiences produce sustained changes in skills and engagement with aged care dentistry.

## 5 Limitations

While this report demonstrates key strengths in the integration of digital denture workflows within RACF placements, several limitations should be acknowledged. The low number of student responses from a single institution may limit generalizability of the findings. The respondent sample was also predominantly male (75.0%), which may not represent the demographic composition of dental student cohorts at other institutions and may further limit the generalizability of the findings. Additionally, voluntary participation may introduce response bias. Although each Likert-scale item offered a full range of response options from “strongly disagree” to “strongly agree,” all items were positively framed and no reverse-worded items were included. Consistent wording was used to make the questionnaire straightforward and easy for participants to understand and complete. However, this design may have increased the risk of acquiescence bias and limited the ability to identify low-engagement or inconsistent responses. Although the questionnaire was completed anonymously, the evaluation involved a placement delivered by members of the research team; therefore, social desirability bias cannot be excluded. Voluntary participation may also have introduced self-selection bias, as students with particularly positive or negative experiences may have been more likely to respond. Accordingly, the largely positive findings should be interpreted cautiously as preliminary, self-reported student perceptions. Furthermore, the evaluation also relied on self-reported student perceptions and did not measure patient-level oral health outcomes or objective changes in access to care. The self-reported data were collected after a short placement period, which may not reflect long-term skill development or sustainability of the educational model. The use of a single-coder-with-review process rather than independent double-coding may have increased the influence of the initial coder's interpretation. Five students did not have the opportunity to complete a digital denture scan and were therefore excluded from analyses of direct scanning experiences. The number of scans completed also varied among participating students, and this unequal exposure may have influenced their confidence and perceptions of workflow usability. Future studies should include larger multi-site cohorts and longitudinal follow-up to assess competency progression.

Despite these limitations, this report highlights the perceived educational value and practical feasibility of digital denture workflows in aged care placements with exposure to oral health needs of the elderly, and interdisciplinary learning and collaboration. These findings provide a strong foundation for future research with larger cohorts and multi-site implementation to further validate the impact of digital dentures in RACF placements. Overall, this innovative model of care has the potential to build much-needed workforce capacity in Special Needs Dentistry, specifically in Aged Care, enhance oral health outcomes, and inform future educational and service delivery frameworks.

The expanding ageing population stresses the need for a workforce equipped to manage complex oral healthcare needs. Placements that provide real-world experience with vulnerable populations may support the development of person-centred and collaborative care skills. Further research should examine the long-term effects of these placements on educational outcomes, workforce preparedness, and engagement in aged care.

## 6 Conclusion

This innovative interdisciplinary mobile dentistry model provided valuable exposure to collaborative practice, mobile dentistry, and digital denture workflows. This enabled students to engage with older adults in aged care environments that are often underrepresented in dental curricula. Students reported increased awareness of geriatric oral healthcare and the challenges faced by older adults. Students reported positive learning experiences and supported the integration of digital denture workflows into placements conducted in complex, real-world aged care environments. However, uneven hands-on scanning opportunities and comparatively low confidence in independent scanning indicate the need for structured rotations, repeated supervised practice, and improved logistical coordination. Inclusion of digital denture workflows can provide meaningful educational value in aged care settings with adequate supervision and support through intersectoral collaboration among education, oral healthcare, and aged care sectors. As this study did not measure resident outcomes or objective changes in access to care, the findings should not be interpreted as evidence of improved prosthodontic access or service efficiency. Larger multicentre and longitudinal studies using validated measures and objective competency assessments are needed to evaluate the educational effects of this placement model and determine its adaptability across different institutional and national contexts.

## Data Availability

The original contributions presented in the study are included in the article/Supplementary Material, further inquiries can be directed to the corresponding author/s.

## Supplementary material

Supplementary Appendix 1 Contains the questionnaire used in this research.
