# Supplementary material for: Mobile dentistry and digital dentures in aged care placements: dental students’ experiences and perceptions
Source: Front Oral Health. 2026 Sep 16;7:1923430. doi: 10.3389/froh.2026.1923430 (PMC13623756; doi:10.3389/froh.2026.1923430)
Supplement: Supplementary file 1 [file Supplementaryfile1.doc]

### Supplementary Table 1. Full distribution of responses to Likert-scale perception items

| **Students’ experience** | **Strongly disagree n (%)** | **Disagree** **n (%)** | **Neutral** **n (%)** | **Agree** **n (%)** | **Strongly agree** **n (%)** |
| --- | --- | --- | --- | --- | --- |
| **Students completing ≥1 scan  (n = 23)** |  |  |  |  |  |
| The demonstration on intraoral scanning was effective. | 0 (0.0) | 0 (0.0) | 2 (8.7) | 11 (47.8) | 10 (43.5) |
| Handling of the intraoral scanner was easy. | 1 (4.3) | 0 (0.0) | 4 (17.4) | 12 (52.2) | 6(26.1) |
| The amount of time required for the scan was acceptable. | 1 (4.3) | 0 (0.0) | 4 (17.4) | 9 (39.1) | 9 (39.1) |
| I am comfortable scanning independently after the training. | 1 (4.3) | 4 (17.4) | 5 (21.7) | 10 (43.5) | 3 (13.0) |
| **Curriculum inclusion—full sample (n = 28)** |  |  |  |  |  |
| Digital denture scanning should be integrated into dental education. | 4 (14.3) | 1 (3.6) | 3 (10.7) | 9 (32.1) | 11 (39.3) |

**Appendix 1**

**Student Questionnaire**

**Quantitative**

**Demographics:**

1.	Age group: ☐<21 years	☐ 21-34 years	☐≥35 years

2.	Gender: ☐ Male ☐ Female ☐ Other ………………….

3.	Did you have any prior experience in scanning digital dentures?

☐ Yes ☐ No

Digital denture scanning experience

4.	How many scans did you perform during the placement?

☐None	☐1–2	☐ 3–5	☐ >6

**If no scans were done, go to Question 10.**

5.	How long did you take to scan a complete denture?

☐ <10 min	☐ 10–20 min	☐ >20 min

| **Students’ experience** | **Strongly disagree** | **Disagree** | **Neutral** | **Agree** | **Strongly agree** |
| --- | --- | --- | --- | --- | --- |
| 6. The demonstration on intraoral scanning was effective. | 1 | 2 | 3 | 4 | 5 |
| 7. Handling of the intraoral scanner was easy. | 1 | 2 | 3 | 4 | 5 |
| 8. The amount of time required for the scan was acceptable. | 1 | 2 | 3 | 4 | 5 |
| 9. I am comfortable scanning independently after the training. | 1 | 2 | 3 | 4 | 5 |
| 10. Digital denture scanning should be integrated into dental education. | 1 | 2 | 3 | 4 | 5 |

**Qualitative questions (Open-ended)**

11.	How did participating in this RACF placement influence your interest in providing oral health care for older adults?

12.	What barriers or challenges did you observe during your placement/when using digital denture workflows in the RACF setting?

13.	What facilitators or benefits did you observe that could support Aged care placement/wider adoption of digital dentures in aged care?

14.	In your opinion, should digital denture training and RACF placements be included as a required part of the dental curriculum? Please explain.

15.	Additional comments: Positive experiences, issues that need improvement, or suggestions for future placements.
